# Effects of Concurrent Strength and Endurance Training on Measures of Physical Fitness in Healthy Middle-Aged and Older Adults: A Systematic Review with Meta-Analysis

**DOI:** 10.1007/s40279-022-01764-2

**Published:** 2022-10-12

**Authors:** Adrian Markov, Lukas Hauser, Helmi Chaabene

**Affiliations:** 1 Division of Training and Movement Sciences, Research Focus Cognition Sciences, University of Potsdam, Am Neuen Palais 10, Bldg. 12, 14469 Potsdam, Germany; 2 Department of Sports and Health Sciences, Faculty of Human Sciences, University of Potsdam, 14469 Potsdam, Germany; 3 High Institute of Sports and Physical Education of Kef, University of Jendouba, 7100 Kef, Tunisia

## Abstract

**Background:**

There is evidence that in older adults the combination of strength training (ST) and endurance training (ET) (i.e., concurrent training [CT]) has similar effects on measures of muscle strength and cardiorespiratory endurance (CRE) compared with single-mode ST or ET, respectively. Therefore, CT seems to be an effective method to target broad aspects of physical fitness in older adults.

**Objectives:**

The aim was to examine the effects of CT on measures of physical fitness (i.e., muscle strength, power, balance and CRE) in healthy middle-aged and older adults aged between 50 and 73 years. We also aimed to identify key moderating variables to guide training prescription.

**Study Design:**

We conducted a systematic review with meta-analysis of randomized controlled trials.

**Data Sources:**

The electronic databases PubMed, Web of Science Core Collection, MEDLINE and Google Scholar were systematically searched until February 2022.

**Eligibility Criteria for Selecting Studies:**

We included randomized controlled trials that examined the effects of CT versus passive controls on measures of physical fitness in healthy middle-aged and older adults aged between 50 and 73 years.

**Results:**

Fifteen studies were eligible, including a total of 566 participants. CT induced moderate positive effects on muscle strength (standardized mean difference [SMD] = 0.74) and power (SMD = 0.50), with a small effect on CRE (SMD = 0.48). However, no significant effects were detected for balance (*p* > 0.05). Older adults > 65 years (SMD = 1.04) and females (SMD = 1.05) displayed larger improvements in muscle strength compared with adults ≤ 65 years old (SMD = 0.60) and males (SMD = 0.38), respectively. For CRE, moderate positive effects (SMD = 0.52) were reported in those ≤ 65 years old only, with relatively larger gains in females (SMD = 0.55) compared with males (SMD = 0.45). However, no significant differences between all subgroups were detected. Independent single training factor analysis indicated larger positive effects of 12 weeks (SMD = 0.87 and 0.88) compared with 21 weeks (SMD = 0.47 and 0.29) of CT on muscle strength and power, respectively, while for CRE, 21 weeks of CT resulted in larger gains (SMD = 0.62) than 12 weeks (SMD = 0.40). For CT frequency, three sessions per week produced larger beneficial effects (SMD = 0.91) on muscle strength compared with four sessions (SMD = 0.55), whereas for CRE, moderate positive effects were only noted after four sessions per week (SMD = 0.58). A session duration of > 30–60 min generated larger improvements in muscle strength (SMD = 0.99) and power (SMD = 0.88) compared with > 60–90 min (SMD = 0.40 and 0.29, respectively). However, for CRE, longer session durations (i.e., > 60–90 min) seem to be more effective (SMD = 0.61) than shorter ones (i.e., > 30–60 min) (SMD = 0.34). ET at moderate-to-near maximal intensities produced moderate (SMD = 0.64) and small positive effects (SMD = 0.49) on muscle strength and CRE, respectively, with no effects at low intensity ET (*p* > 0.05). Finally, intra-session ST before ET produced larger gains in muscle strength (SMD = 1.00) compared with separate sessions (SMD = 0.55), whereas ET and ST carried out separately induced larger improvements in CRE (SMD = 0.58) compared with intra-session ET before ST (SMD = 0.49).

**Conclusions:**

CT is an effective method to improve measures of physical fitness (i.e., muscle strength, power, and CRE) in healthy middle-aged and older adults aged between 50 and 73 years, regardless of sex. Results of independent single training factor analysis indicated that the largest effects on muscle strength were observed after 12 weeks of training, > 30–60 min per session, three sessions per week, higher ET intensities and when ST preceded ET within the same session. For CRE, the largest effects were noted after 21 weeks of training, four sessions per week, > 60–90 min per session, higher ET intensities and when ET and ST sessions were performed separately. Regarding muscle power, the largest effects were observed after 12 weeks of training and > 30–60 min per session.

## Key Points

**Table:** 

Concurrent training is an effective method to improve measures of physical fitness (i.e., muscle strength, power, and cardiorespiratory endurance) in healthy adults aged between 50 and 73 years, regardless of sex.
Concurrent training resulted in larger effects on muscle strength and cardiorespiratory endurance in females compared with males.
Results of independent single training factor analysis indicated that the largest effects on muscle strength were observed after 12 weeks of training, > 30–60 min per session, three sessions per week, higher endurance training intensities, and intra-session strength before endurance training. For cardiorespiratory endurance, the largest effects were noted after 21 weeks of training, four sessions per week, > 60–90 min per session, higher endurance training intensities, and separate endurance and strength training sessions.

## Introduction

The absolute number of older adults around the world is sharply increasing [1], making ageing a key policy issue for national and international health organizations. In 2015, the World Health Organization (WHO) published the “World Report on Ageing and Health,” emphasizing the need to take public health action and outlining healthy ageing as more than just the absence of disease but as a “process of developing and maintaining the functional ability that enables well-being in older age” [1]. Within this holistic concept, physical activity is the most important among the behavioral and lifestyle factors and a central component of primary and tertiary prevention [2]. The positive effects of physical activity on health (e.g., preventing cardiovascular disease [3] and type 2 diabetes [4], reducing the risk of stroke [5, 6], breast and colon cancer [2], reducing all-cause mortality risk [7]) were previously promoted by global health organizations [8–10]. It is worth noting that the WHO attributed 6% of deaths worldwide to physical inactivity making it the fourth leading risk factor for death, globally [9]. Additionally, recent findings indicate a worldwide trend towards insufficient physical activity [11, 12], which emerges substantially with ascending age [2, 13]. This applies particularly to older adults (≥ 60 years), with a median prevalence of up to 54.6% [9].

The biological process of ageing is characterized by multifactorial, morphological and functional changes [14–16]. More specifically, ageing is associated with a decline in the level of physical fitness [17–19], resulting in adverse outcomes such as impaired mobility, increased risk for falls [20] and reduced quality of life [21–23]. These alterations are known to be more prevalent in older populations [24], but evidence indicates that the decrease in muscle mass and function (i.e., muscle strength and power) starts from ~ 40 years onwards [24, 25] and begins to be visible at ~ 50 years of age [24–27]. Additionally, earlier studies indicated that the level of physical fitness tracks from middle-age to older adult age [28]. This implies that the level of physical fitness in middle-age can predict physical performance in later life, indicating that earlier training interventions at ~ 50 years can result in positive long-term effects. Therefore, maintaining a high level of physical fitness is of utmost importance in both middle-aged and older adults. The mechanisms underlying age-related alterations in physical fitness level and motor control are multifactorial, yet not fully understood [15]. The available evidence indicated that ageing negatively affects human skeletal muscle architecture [16], muscle mass and function (i.e., sarcopenia) [29–31] as well as neural processes [14, 32, 33]. These adverse effects result in impairments in instrumental activities of daily living [34] and increase the risk of functional dependency and frailty [34, 35].

The available recommendations for exercise training and physical activity for older adults comprise endurance training (ET) and strength training (ST) [10, 22, 23]. In this regard, it is well-established that ST and ET induce specific adaptations pertaining to muscle architecture [36–38], neural factors [39, 40] or energy metabolism [41, 42]. Specifically, ample evidence indicated that ST induces beneficial effects on muscle protein synthesis [43], muscle cross-sectional area (CSA) [44–46] and neural excitability [47], all of which lead to increased muscle strength [48, 49], muscle power [50, 51] and rate of force development [52]. ET on the other hand primarily activates mitochondrial biogenesis and angiogenesis (i.e., formation of new capillary blood vessels from pre-existing ones), which in turn improve cardiovascular functions and muscle metabolism [53–55]. As such, the combination of both ST and ET (i.e., concurrent training [CT]) could be an effective strategy to improve diverse measures of physical fitness (e.g., muscle strength, muscle power, cardiorespiratory endurance [CRE]) in older adults [22, 56, 57].

Previous descriptive reviews have recommended CT to promote health and counteract ageing-related functional declines in older populations [58, 59]. In general, there is strong evidence suggesting that in older adults, CT induces similar adaptations in muscle strength and muscle power compared with single-mode ST [57, 60, 61]. Likewise, it has been shown that CT in older adults leads to similar improvements in CRE (i.e., peak oxygen uptake [$\dot{V}{\text{O}}_{{2{\text{peak}}}}$], maximal aerobic cycle ergometer workload [*W*_max_]) compared with single-mode ET [57, 62, 63]. Therefore, CT appears to be an effective approach allowing the development of both muscle strength and CRE in older adults. To the best of our knowledge, there is only one systematic review with meta-analysis [64] addressing the effects of CT on measures of CRE (e.g., $\dot{V}{\text{O}}_{{2{\text{peak}}}}$) and functional performance (i.e., Timed Up  & Go and 30-s chair stand) in adults aged over 50 years. However, this review presents several methodological shortcomings [64]. For instance, the authors included non-randomized trials and studies with active control groups, biasing the identification of the true effects of CT. Additionally, studies that combined CT alongside other training methods (e.g., balance) were included. Moreover, the authors did not account for moderating factors such as age, sex or training variables. All these shortcomings highlight the need for future research to draw more robust conclusions. Furthermore, the effects of CT on measures of muscle strength, muscle power and balance in older adults are yet to be meta-analyzed.

Therefore, this systematic review and meta-analysis aimed (1) to examine the effects of CT on measures of physical fitness (i.e., muscle strength, power, balance and CRE) in healthy middle-aged and older adults aged between 50 and 73 years and (2) to quantify the moderating effects of age, sex and training variables (i.e., intervention duration, training frequency, session duration, CT configuration, ST intensity, ET intensity) to help inform training prescription.

## Methods

This systematic review and meta-analysis was conducted according to the Preferred Reporting Items for Systematic Reviews and Meta-Analyses (PRISMA) statement [65, 66] and was registered in the International Prospective Register of Systematic Reviews (PROSPERO) database on 5 July 2020 under the registration number “CRD42020188618”.

### Literature Search

**Fig. 1:**
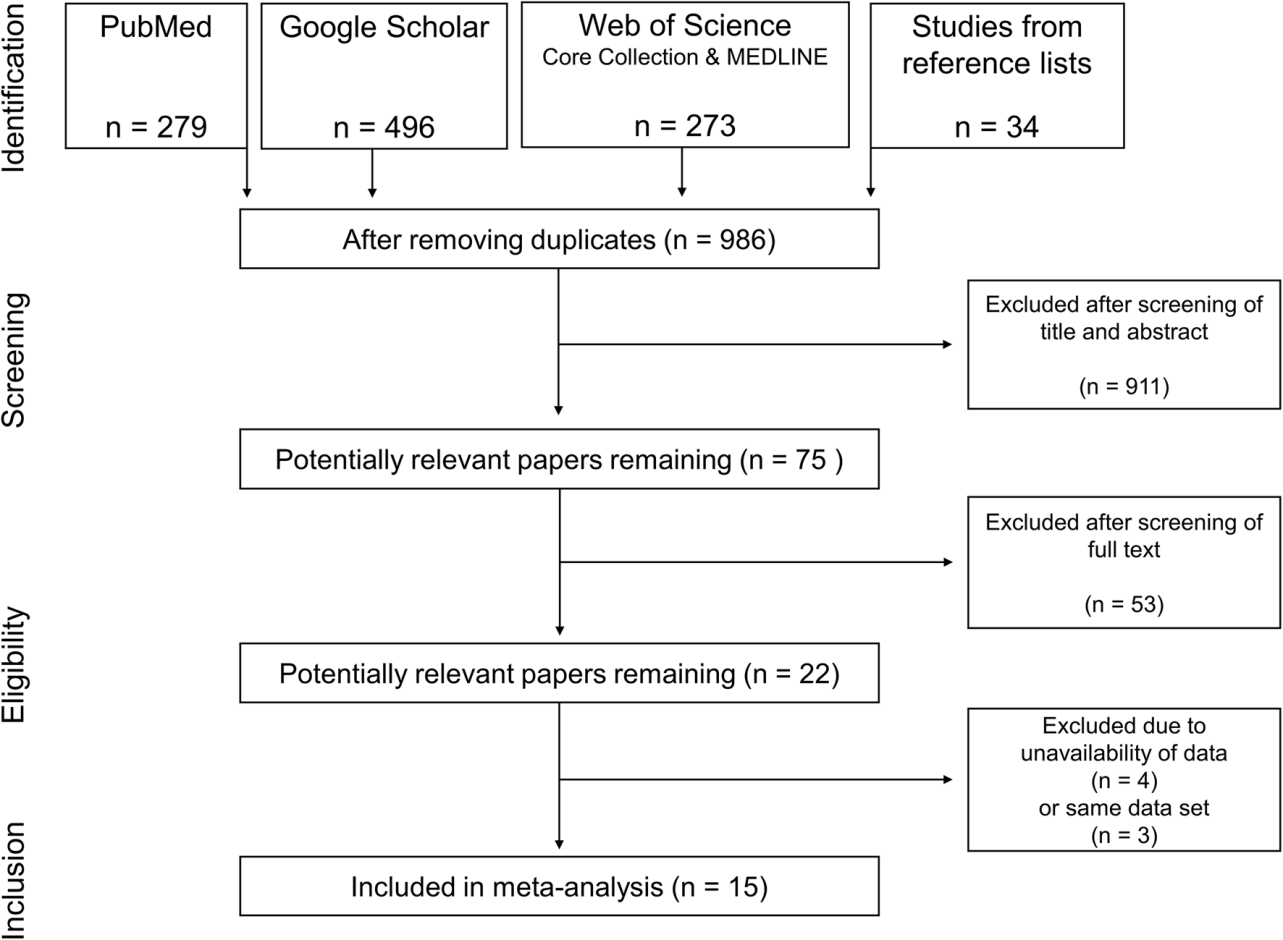
PRISMA flow chart illustrating the different phases of the search and study selection. *PRISMA* Preferred Reporting Items for Systematic Reviews and Meta-Analyses

A systematic literature search was conducted using the electronic bibliographic databases PubMed, Web of Science Core Collection, MEDLINE and Google Scholar with no date restrictions up to February 2022. Keywords were collected through experts’ opinions, literature review and controlled vocabulary (e.g., Medical Subject Headings [MeSH]). The search was limited to peer-reviewed, randomized controlled studies written in English. A Boolean search syntax was applied using the operators “AND,” “OR” and “NOT.” The following syntax is an example of a PubMed search: ("strength training" OR "resistance training" OR "endurance training" OR “aerobic training” OR “cardiorespiratory endurance”) AND (training OR exercise OR concurrent*) AND (old OR elderly OR seniors or “older adults”) AND (“physical fitness” OR strength OR power OR endurance OR “aerobic capacity” OR “motor performance”) NOT (rehabilitation OR patients OR disease* OR pain OR injury OR "multiple sclerosis" OR cancer OR diabetes OR obes* OR dementia). Search results were screened by two authors (AM, LH). First, titles of all relevant articles were screened. Thereafter, abstracts and finally full texts were examined to confirm the inclusion. Reference lists of eligible articles were manually searched to identify further potentially relevant publications. If a study did not fulfill all criteria, the respective exclusion criterion was documented and the study was not considered for further analysis. In the case of disagreement between the two authors, a third co-author (HC) was consulted. An overview of the screening process is outlined in Fig. 1.

### Eligibility Criteria

Following the PRISMA statement, a PICOS (participants, intervention, comparators, study outcomes, and study design) approach was used to rate studies for eligibility [65]. Inclusion criteria were applied as displayed in Table 1.

**Table 1:** Study selection

Category	Inclusion criteria	Exclusion criteria
Population	Healthy adults aged ≥ 50 years, irrespective of sex and level of physical activity	Individuals with adverse health events (e.g., diabetes, sarcopenia, asthma, hypertension) or outside the preferred age range
Intervention	Concurrent training interventions (i.e., a combination of ST and ET)	Single-mode training interventions (e.g., single-mode ST or ET)
Comparator	Passive control group	Absence of a control group, active controls
Outcome	Measures of physical fitness (i.e., muscle strength, cardiorespiratory endurance, muscle power, balance)	Lack of baseline and/or follow-up data
Study design	Randomized controlled trials	Non-randomized controlled trials

*ET* endurance training, *ST* strength training

### Data Extraction

Data from the included studies were extracted into a template created with Microsoft Excel [67] by one author (LH) and verified by a second one (AM). The source (name of the first author and year of publication), participant characteristics (age, sex, number), training variables (intervention duration, frequency, session duration, intensity) and main outcome(s) of the included studies were extracted. In the case of no agreement regarding data extraction, a third co-author (HC) was consulted for clarification. To compute effect sizes, baseline and follow-up means and standard deviations (SDs) for measures of physical fitness of both the intervention and control groups were extracted. If the required data (i.e., means and SD) were not reported in the article or were presented in an inappropriate format for data extraction, the corresponding authors were contacted and kindly asked to provide the missing values. If the relevant data were not available, the respective study was excluded. In the case of multiple tests being used for the same measure of physical fitness, protocols with superior criterion validity [68] were selected. The extracted data were coded as outlined in Table 2. The characteristics of the included studies are presented in Table 3.

**Table 2:** Study coding

Outcome categories	Measure
Muscle strength	Maximal isokinetic torque of the knee extensors
	Maximal isometric force of knee extensors One-repetition maximum of knee extensors
Muscle power	Muscle power of knee extensors
	Rate of force development of knee extensors
	Countermovement jump height
	Squat jump height
	Squat jump power
Cardiorespiratory endurance	Maximal oxygen uptake (V̇O_2peak_ or $\dot{V}{\text{O}}_{2\max }$)
	Maximal aerobic workload
Balance*	Center of pressure surface area or distance Timed-up-and-go test

$\dot{V}$*O*_*2max*_ maximal oxygen uptake, $\dot{V}$*O*_*2peak*_ peak oxygen uptake

^*^ Balance was used as an umbrella term to describe both static and dynamic balance

**Table 3:** Characteristics of the included studies

References	Training moderator variables						Comp	Sex	*N*	Experimental (pre-test)		Experimental (post-test)		*N*	Control (pre-test)		Control (post-test)	
	tvol	tfre	sdur	Intensity ST	Intensity ET	tseq				Mean	SD	Mean	SD		Mean	SD	Mean	SD
Abbasi et al. [88]	8	3	na	Moderate-to-hard	Low	SE	MS	Men	8	46.80	75.89	54.90	87.01	8	43.10	62.29	43.10	25.02
Amaro-Gahete et al. [89]	12	3	70	very-light-to-light	Low	ES	MS	Men	8	337.50	37.70	365.38	31.15	4	314.90	70.10	347.83	40.70
	12	3	70	Very-light-to-light	Low	ES	CRE	Men	8	35.00	6.30	38.49	8.18	4	33.10	3.30	32.56	4.28
	12	2	30	Very-light-to-light	Moderate-to-high	ES	MS	Men	9	407.70	52.50	441.67	56.56	4	314.90	70.10	347.83	40.70
	12	2	30	Very-light-to-light	Moderate-to-high	ES	CRE	Men	9	33.10	4.60	36.58	4.90	4	33.10	3.30	32.56	4.28
	12	3	70	Very-light-to-light	Low	ES	MS	Women	9	212.80	45.90	238.00	67.51	6	204.30	39.70	214.63	27.42
	12	3	70	Very-light-to-light	Low	ES	CRE	Women	9	28.70	4.40	32.00	3.94	6	26.10	3.70	26.75	3.75
	12	2	30	Very-light-to-light	Moderate-to-high	ES	MS	Women	9	198.40	30.30	229.89	28.55	6	204.30	39.70	214.63	27.42
	12	2	30	Very-light-to-light	Moderate-to-high	ES	CRE	Women	9	30.10	7.50	33.11	8.44	6	26.10	3.70	26.75	3.75
Campos et al. [90]	12	3	60	Moderate-to-hard	Moderate-to-high	ES	MS	Women	5	66.00	19.49	74.60	33.50	1.5	49.30	9.00	47.00	13.90
	12	3	60	Moderate-to-hard	Moderate-to-high	SE	MS	Women	5	61.00	29.70	63.60	17.40	1.5	49.30	9.00	47.00	13.90
Figueroa et al. [91]	12	3	40	Moderate-to-hard	Low	SE	MS	Women	12	38.30	1.10	43.30	1.70	12	38.30	1.50	38.80	1.50
Haykowsky et al. [92]	12	3	30–60	Moderate-to-hard	Low	ES	MS	Women	7	169.10	41.00	263.60	89.00	7	20.50	36.30	203.80	43.20
	12	3	30–60	Moderate-to-hard	Low	ES	MP	Women	7	96.00	29.00	123.00	25.00	7	95.00	15.00	95.00	13.00
	12	3	30–60	Moderate-to-hard	Low	ES	CRE	Women	7	59.00	20.00	67.00	14.00	7	63.00	13.00	57.00	10.00
Holviala et al. [63]	21	4	60–90	Moderate-to-hard	Moderate-to-high	sep	MS	Men	11	707.00	132.00	792.00	116.00	9	682.00	130.00	732.00	119.00
	21	4	60–90	Moderate-to-hard	Moderate-to-high	sep	MP	Men	11	1601.00	434.00	1611.00	428.00	9	1392.00	258.00	1463.00	422.00
	21	4	60–90	Moderate-to-hard	Moderate-to-high	sep	CRE	Men	11	32.90	4.20	35.70	4.30	9	33.50	4.20	34.70	6.40
Holviala et al. [98]	21	4	60–90	Moderate-to-hard	Moderate-to-high	sep	MP	Men	31	724.00	152.50	797.40	176.50	21	655.90	129.60	650.20	147.30
	21	4	60–90	Moderate-to-hard	Moderate-to-high	sep	B	Men	31	680.90	135.90	616.60	98.80	21	733.80	270.30	665.10	167.90
Karavirta et al. [60]	21	4	60–90	Moderate-to-hard	Moderate-to-high	sep	MS	Men	29	2776.00	668.00	3314.00	815.00	16	2642.00	389.00	2793.00	473.00
	21	4	60–90	Moderate-to-hard	Moderate-to-high	sep	MP	Men	29	32.50	4.20	35.70	5.10	16	34.80	5.50	34.80	6.00
Karavirta et al. [86]	21	4	60–90	Moderate-to-hard	Moderate-to-high	sep	CRE	Men	30	2000.00	477.00	2250.00	555.00	16	1843.00	440.00	1843.00	411.00
Karavirta et al. [87]	21	4	60–90	Moderate-to-hard	Moderate-to-high	sep	MS	Women	23	1977.00	498.00	2446.00	793.00	17	1882.00	278.00	1970.00	239.00
	21	4	60–90	Moderate-to-hard	Moderate-to-high	sep	CRE	Women	23	26.80	4.90	31.10	5.00	17	26.60	6.10	26.80	5.80
Libardi et al. [93]	12	4	40	Moderate-to-hard	Moderate-to-high	sep	MS	Both	8	168.13	53.18	231.63	81.48	3.5	223.57	81.23	202.50	84.19
	12	4	40	Moderate-to-hard	Moderate-to-high	sep	CRE	Both	8	23.52	5.34	25.74	6.13	3.5	22.51	3.57	22.26	5.06
	12	4	40	Moderate-to-hard	Moderate-to-high	sep	MS	Both	10	166.90	72.58	200.33	73.93	3.5	223.57	81.23	202.50	84.19
	12	4	40	Moderate-to-hard	Moderate-to-high	sep	CRE	Both	10	24.15	3.94	26.42	4.80	3.5	22.51	3.57	22.26	5.06
Takeshima et al. [94]	12	3	50	Moderate-to-hard	Low	ES	MS	Both	18	128.40	39.90	140.30	42.50	17	124.20	36.80	121.80	35.60
	12	3	50	Moderate-to-hard	Low	ES	CRE	Both	18	1.36	0.25	1.56	0.28	17	1.32	0.29	1.37	0.37
Timmons et al. [95]	12	3	40	Moderate-to-hard	Moderate-to-high	SE	MS	Both	21	114.10	30.70	165.80	40.20	21	99.50	31.40	98.80	33.30
	12	3	40	Moderate-to-hard	Moderate-to-high	SE	B	Both	21	6.49	0.71	5.31	0.75	21	7.65	1.46	7.48	1.99
Wilhelm et al. [96]	12	2	40–60	Moderate-to-hard	Moderate-to-high	SE	MS	Men	12	26.30	6.30	29.90	6.50	6.5	24.70	6.20	24.80	5.70
	12	2	40–60	Moderate-to-hard	Moderate-to-high	SE	MP	Men	12	254.00	54.00	310.00	80.00	6.5	265.00	92.00	258.00	87.00
	12	2	40–60	Moderate-to-hard	Moderate-to-high	SE	CRE	Men	12	23.70	7.80	25.80	7.20	6.5	21.00	2.70	21.40	2.60
	12	2	40–60	Moderate-to-hard	Moderate-to-high	SE	B	Men	12	4.96	0.65	4.98	0.61	6.5	4.75	0.62	4.73	0.73
	12	2	40–60	Moderate-to-hard	Moderate-to-high	ES	MS	Men	11	27.00	6.50	31.40	6.20	6.5	24.70	6.20	24.80	5.70
	12	2	40–60	Moderate-to-hard	Moderate-to-high	ES	MP	Men	11	249.00	68.00	306.00	81.00	6.5	265.00	92.00	258.00	87.00
	12	2	40–60	Moderate-to-hard	Moderate-to-high	ES	CRE	Men	11	23.30	4.00	24.90	5.80	6.5	21.00	2.70	21.40	2.60
	12	2	40–60	Moderate-to-hard	Moderate-to-high	ES	B	Men	11	5.19	1.30	4.98	0.95	6.5	4.75	0.62	4.73	0.73
Yoon et al. [97]	12	3	60	Moderate-to-hard	Low	SE	MS	Women	10	156.78	33.71	181.93	32.80	5	166.27	47.57	163.64	47.45
	12	3	60	Moderate-to-hard	Low	SE	CRE	Women	10	27.95	4.91	27.43	6.90	5	27.09	6.41	27.38	5.53
	12	3	60	Moderate-to-hard	Low	SE	B	Women	10	3.97	0.56	3.47	0.35	5	3.83	0.28	3.91	0.37

*B* balance, *CRE* cardiorespiratory endurance, *ET* endurance training, *idur* intervention duration, *MP* muscle power, *MS* muscle strength, *N* number of participants, *sdur* session duration, *sep* separate sessions, *ST* strength training, *tfre* training frequency

### Methodological Quality and Risk of Bias

**Fig. 2:**
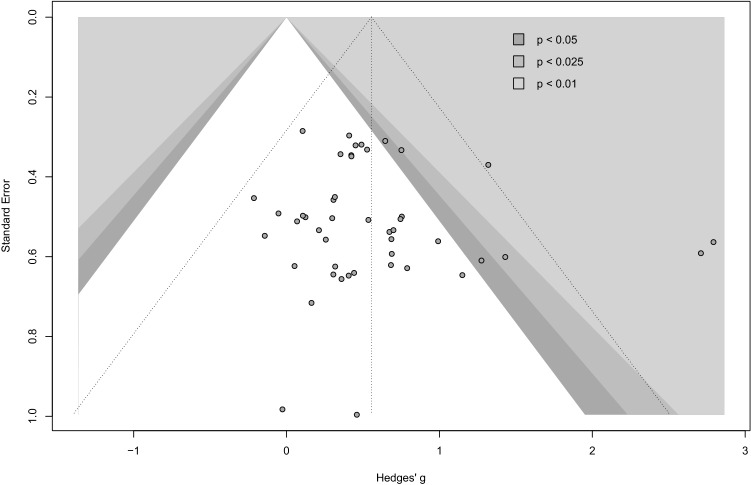
Funnel plot: risk of bias assessment

We used the Physiotherapy Evidence Database (PEDro) scale to appraise the methodological quality and to estimate the potential risk of bias of the eligible studies. The internal study validity and the presence of statistical replicable information were rated on a scale from 0 (high risk of bias) to 10 (low risk of bias), with a score ≥ 6 representing a threshold for studies with low risk of bias [69]. Further, contour-enhanced funnel plots were generated by plotting the effect sizes (Hedges’ *g*) of each study against the respective standard error. To quantify the funnel plot asymmetry and to estimate the risk of publication bias, Egger’s test of the intercept was used [70]. Results of the risk of bias assessment are displayed in Table 4 and Fig. 2.

**Table 4:** Study quality

Studies	PEDRo Scale Items*											PEDro Score
	1	2	3	4	5	6	7	8	9	10	11	
Abbasi et al. [88]	1	1	0	1	0	0	0	0	0	1	1	4
Amaro-Gahete et al. [89]	1	1	0	1	0	0	1	0	0	1	1	5
Campos et al. [90]	1	1	0	1	0	0	0	1	0	1	1	5
Figueroa et al. [91]	1	1	0	1	0	0	0	1	0	1	1	5
Haykowsky et al. [92]	0	1	0	1	0	0	1	1	0	1	1	6
Holviala et al. [63]	1	1	0	1	0	0	0	1	0	1	1	5
Holviala et al. [98]	1	1	0	1	0	0	0	1	0	1	1	5
Karavirta et al. [60]	1	1	0	1	0	0	0	1	0	1	0	4
Karavirta et al. [86]	1	1	0	1	0	0	0	0	0	1	0	3
Karavirta et al. [87]	1	1	0	1	0	0	0	1	0	1	1	5
Libardi et al. [93]	1	1	0	1	0	0	0	0	0	1	0	3
Takeshima et al. [94]	0	1	0	1	0	0	1	0	0	1	1	5
Timmons et al. [95]	1	1	0	1	0	0	1	1	0	1	1	6
Wilhelm et al. [96]	1	0	0	1	0	0	0	0	0	1	1	3
Yoon et al. [97]	0	1	0	1	0	0	0	0	0	1	1	4
									Median score =			5

*PEDro* Physiotherapy Evidence Database

PEDro scale items: (1) eligibility criteria were specified; (2) participants were randomly allocated to groups (in a crossover study, participants were randomly allocated an order in which treatments were received); (3) allocation was concealed; (4) the groups were similar at baseline regarding the most important prognostic indicators; (5) there was blinding of all participants; (6) there was blinding of all therapists who administered the therapy; (7) there was blinding of all assessors who measured at least one key outcome; (8) measures of at least one key outcome were obtained from more than 85% of the participants initially allocated to groups; (9) all participants for whom outcome measures were available received the treatment or control condition as allocated or, where this was not the case, data for at least one key outcome were analyzed by “intention to treat”; (10) the results of between-group statistical comparisons are reported for at least one key outcome; (11) the study provides both point measures and measures of variability for at least one key outcome

### Statistical Analyses

To calculate the effects of CT on measures of physical fitness, weighted between-study standardized mean differences (SMDs) were calculated using the equation $\mathrm{SMD}= \frac{{m}_{1}- {m}_{2}}{{s}_{\mathrm{pooled}}}$, with *m*_1_ representing the mean pre/post-test value of the intervention group, *m*_2_ the mean pre/post-test value of the control group and *s*_pooled_ the pooled SD. Following Hedges and Olkin [71], SMDs were adjusted for the respective sample size using the factor $1- \frac{3}{4N-9}$, with *N* representing the total sample size. If there was more than one intervention group, the control group was divided proportionally by the number of experimental groups to facilitate comparison between all participants [68]. The SMD values were presented alongside 95% confidence intervals (CIs), and effects were interpreted as trivial (SMD < 0.20), small (0.2 ≤ SMD < 0.50), moderate (0.50 ≤ SMD < 0.80) or large (SMD ≥ 0.80) [72]. Of note, reductions in the measures of balance (i.e., center of pressure distance or area, timed-up-and-go performance) were reported as positive values for better readability. To estimate the overall effects of CT on measures of physical fitness, we pooled effect sizes using a random-effects pooling model approach [73]. We used this approach because we assumed that our data derived from a heterogeneous population and interventions vary in certain characteristics. We estimated the variance of the distribution of true effect sizes denoted by *τ*^2^, choosing the Sidik-Jonkman estimator [74] with Hartung-Knapp adjustment. This method has been shown to produce more robust estimates and outperforms the often-used DerSimonian-Laird estimator [75], especially when the number of studies is small and there is substantial heterogeneity [74, 76]. At least three studies had to be included to pool the data and calculate the main effects of CT on each measure of physical fitness [68]. We assessed the level of between-study heterogeneity using Higgin’s and Thompson’s *I*^2^ [77], which displays the amount of variability not caused by sampling error [76]. The level of between-study heterogeneity was interpreted as low (*I*^2^ < 25%), moderate (25% ≤ *I*^2^ < 50%), high (50% ≤ *I*^2^ < 75%) or considerably high (*I*^2^ ≥ 75%) [68, 78]. Further, a multivariate random-effects meta-regression was conducted to verify if any of the training variables (i.e., intervention duration, training frequency, session duration, training order, ST intensity, ET intensity) predicted the effects of CT on measures of physical fitness. As recommended, we only computed meta-regression for covariates denoted by at least ten studies [68, 73]. The level of statistical significance was set at *p* ≤ 0.05. All analyses were conducted using R (v. 3.6.0) [79], using the packages “meta” [80] and “metaphor” [81].

### Subgroup and Single-Factor Analyses

Subgroup analyses were computed for the factors sex (male vs. female) and age (≤ 65 vs. > 65). In addition, single-factor analyses for training variables were conducted. For that, we analyzed the effects of training frequency (i.e., 2 vs. 3 vs. 4 sessions per week), session duration (i.e., > 30–60 min vs. > 60–90 min), total intervention duration (i.e., 12 vs. 21 weeks), CT configuration (i.e., intra-session ST prior to ET vs. intra-session ET prior to ST vs. separate days) and training intensity for ET (i.e., low vs. moderate-to-near maximal). According to the American College of Sports Medicine [8], ET intensities below 70% of the $\dot{V}{\text{O}}_{{2{\text{peak}}}}$, below 70% of the maximum heart rate (HR_max_) or below the anaerobic threshold were interpreted as “low,” while intensities above 70% $\dot{V}{\text{O}}_{{2{\text{peak}}}}$, 70% HR_max_ or the anaerobic threshold were considered “moderate-to-near maximal” [8].

## Results

### Study Selection

Figure 1 illustrates the systematic search process. The search strategy yielded a total of 1048 hits. The reference list search of the included studies provided 34 further studies. After screening study titles and eliminating duplicates, 986 potentially eligible studies were identified. Following the abstract examination, 75 studies remained. After reviewing the full texts, 53 studies were excluded. Out of the remaining 22 studies, seven studies were further excluded due to unavailable data [57, 62, 82, 83] or because of reporting outcomes and datasets [61, 84, 85], which were already presented in other studies [60, 86, 87]. Finally, 15 studies were eligible for inclusion in this meta-analysis (Table 3).

### Description of the Included Studies

The 15 eligible studies included an overall sample size of 566 participants, with a mean age ranging from 50 to 73.5 years (mean 61.0 ± 5.9 years). Out of the studies that reported sex distribution, 188 were females and 363 males, with 332 participants (228 males, 104 females) receiving the training intervention and 219 (135 males, 84 females) serving as controls. The characteristics of the included studies are summarized in Table 3. Twelve out of the 15 studies provided data with respect to the effects of CT on muscle strength [63, 86–97]. Five studies investigated the effects of CT on muscle power [60, 63, 92, 96, 98], nine studies on CRE [63, 86, 87, 89, 92–94, 96, 97] and four studies on balance [95–98]. Intervention duration ranged from 8 to 21 weeks, and training frequency varied from two to four sessions per week, with CT sessions lasting from 30 to 90 min. A more thorough description of the study characteristics is provided in Table 3.

### Methodological Quality and Risk of Bias Assessment

The median PEDro score across the included studies was 5 (range 3–6), with only two studies [92, 95] reaching the score of 6 (Table 4). Results from the risk of bias assessment using funnel plot asymmetry are displayed in Fig. 2. Egger’s test of the intercept provided no evidence for funnel plot asymmetry and potential publication bias (*p* > 0.05).

### Main Effects

**Fig. 3:**
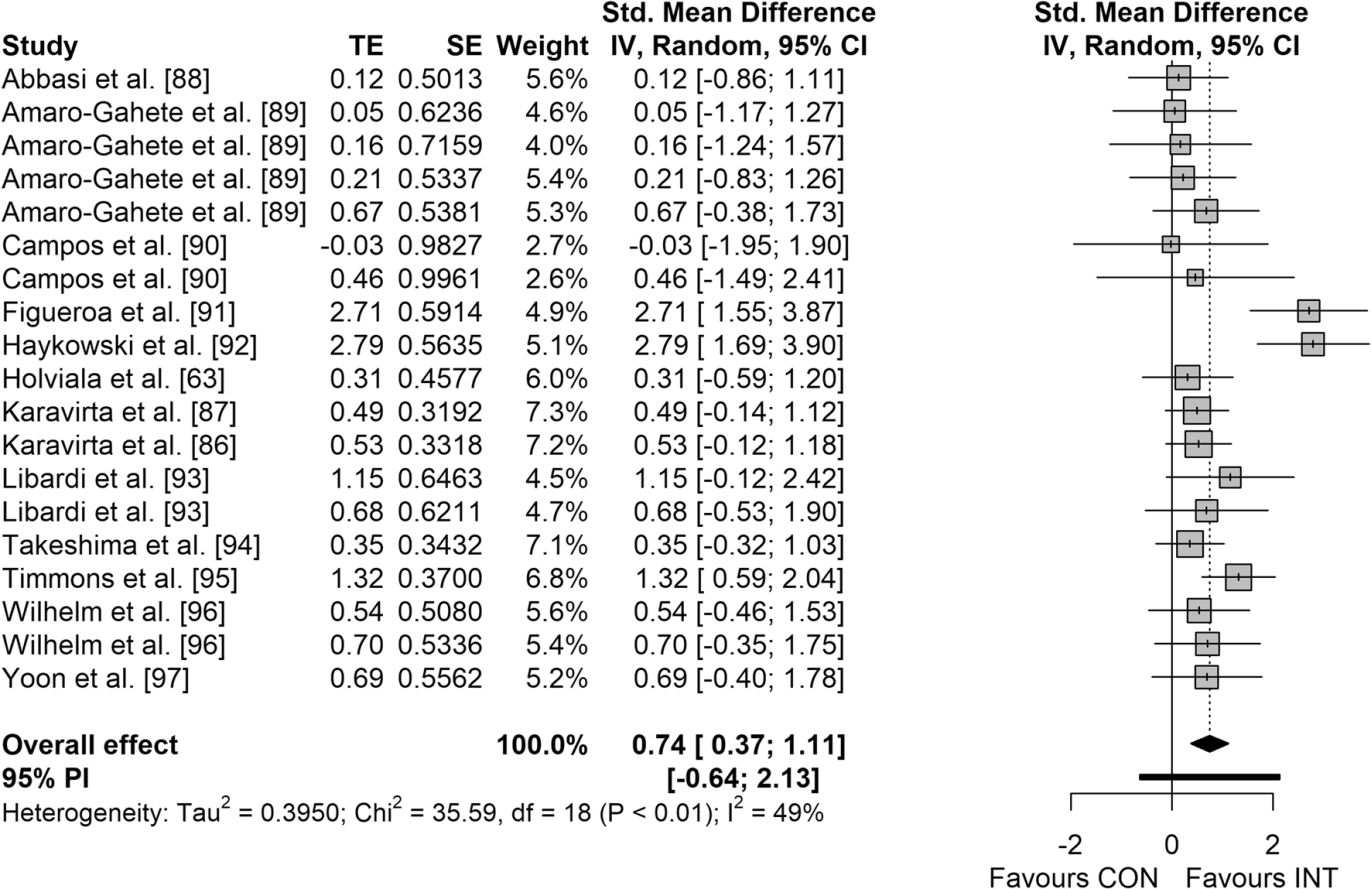
Forest plot for the overall effect of concurrent training on measures of muscle strength. *CI* confidence interval, *CON* control, *df* degrees of freedom, *INT* intervention, *IV* inverse variance, *PI* prediction interval, *SE* standard error of the effect size, *Std.* standard, *TE* calculated effect size

**Fig. 4:**
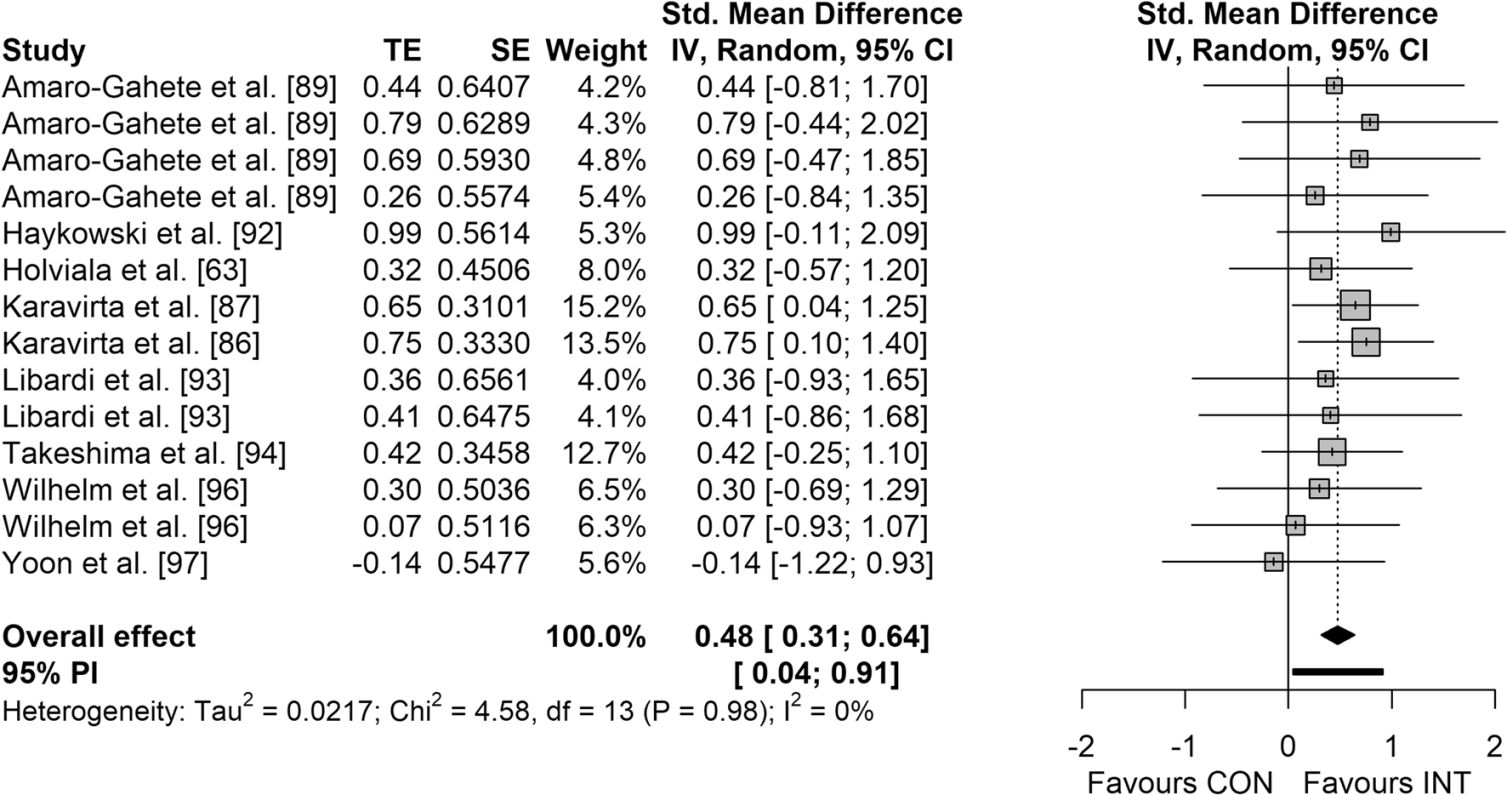
Forest plot for the overall effect of concurrent training on measures of cardiorespiratory endurance. *CI* confidence interval, *CON* control, *df* degrees of freedom, *INT* intervention, *IV* inverse variance, *PI* prediction interval, *SE* standard error of the effect size, *Std.* standard, *TE* calculated effect size

**Fig. 5:**
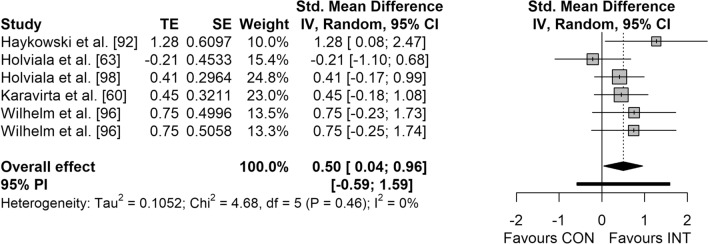
Forest plot for the overall effect of concurrent training on measures of muscle power. *CI* confidence interval, *CON* control, *df* degrees of freedom, *INT* intervention, *IV* inverse variance, *PI* prediction interval, *SE* standard error of the effect size, *Std.* standard, *TE* calculated effect size

**Fig. 6:**
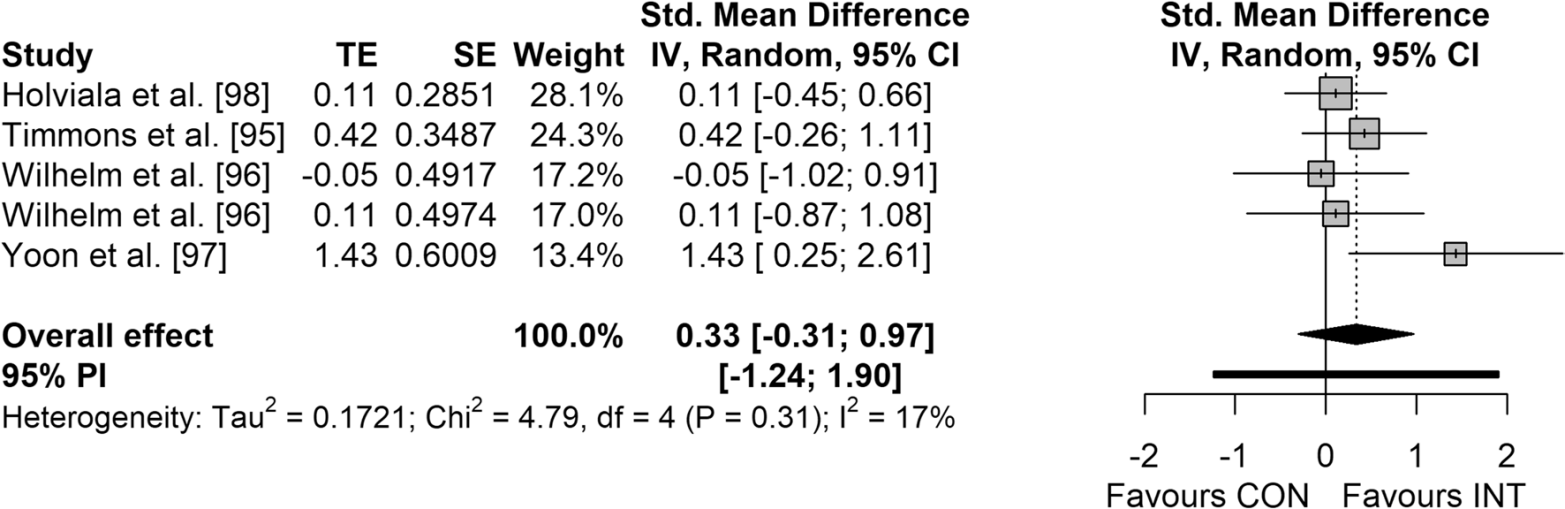
Forest plot for the overall effect of concurrent training on measures of balance. *CI* confidence interval, *CON* control, *df* degrees of freedom, *INT* intervention, *IV* inverse variance, *PI* prediction interval, *SE* standard error of the effect size, *Std.* standard, *TE* calculated effect size

Figures 3, 4, 5 and 6 display the overall effects of CT on measures of physical fitness in middle-aged and older adults. CT induced moderate effects on muscle strength (SMD = 0.74 [95% CI 0.37–1.11]; *p* < 0.001; *I*^2^ = 49.4%) (Fig. 3) and power (SMD = 0.50 [95% CI 0.04–0.96]; *p* = 0.037; *I*^2^ = 0.0%) (Fig. 5). Additionally, a small effect was observed for CRE (SMD = 0.48 [95% CI 0.31–0.64]; *p* < 0.001; *I*^2^ = 0.0%) (Fig. 4). However, no significant effects were detected for balance (SMD = 0.33 [95% CI − 0.31 to 0.97]; *p* = 0.221; *I*^2^ = 16.5%) (Fig. 6).

### Results of Subgroup Analyses

The results of the subgroup analyses are displayed in Table 5. For measures of muscle strength, CT induced moderate effects in middle-aged and older adults aged ≤ 65 years (SMD = 0.60 [95% CI 0.19–1.01]; *p* < 0.05; *I*^2^ = 29.7%), with a large effect in adults aged > 65 years (SMD = 1.04 [95% CI 0.07–2.01]; *p* < 0.05; *I*^2^ = 68.4%). Regarding sex, CT resulted in large effects in females (SMD = 1.05 [95% CI 0.13–1.98]; *p* < 0.05; *I*^2^ = 71.7%), with small effects in males (SMD = 0.38 [95% CI 0.18–0.59]; *p* < 0.05; *I*^2^ = 0.0%). However, no statistically significant difference between subgroups was detected (*p* > 0.05).

**Table 5:** Results of overall, subgroup and single training factor analyses

	Muscle strength			Muscle power			Cardiorespiratory endurance			Balance		
	SMD [95% CI]	S (I)	N (C)	SMD [95% CI]	S (I)	N (C)	SMD [95% CI]	S (I)	N (C)	SMD [95% CI]	S (I)	N (C)
Overall	**0.74 [0.37; 1.11]**	12 (19)	235 (158)	**0.50 [0.04; 0.96]**	5 (6)	102 (66)	**0.48 [0.31; 0.64]**	9 (14)	184 (114)	0.33 [– 0.31; 0.97]	4 (5)	85 (65)
Age	*p* = 0.30			*p* = 0.049			*p* = 0.56			*p* = 0.29		
≤ 65	**0.60** [0.19; 1.01]	8 (13)	162 (103)	0.36 [– 0.19; 0.91]*	4 (4)	83 (59)	**0.52** [0.35; 0.69]	5 (10)	137 (80)	**0.11** [0.10; 0.11]	2 (2)	42 (34)
> 65	**1.04** [0.07; 2.01]	6 (6)	73 (71)	0.97 [– 2.29; 4.22]*	2 (2)	19 (20)	0.39 [– 0.27; 1.05]	4 (4)	47 (47)	0.54 [– 1.21; 2.29]	3 (3)	43 (44)
Sex	*p* = 0.06			*p* = 0.17			*p* = 0.71			*p* = 0.049		
Male	**0.38** [0.18; 0.59]	5 (7)	98 (55)	0.41 [– 0.02; 0.84]	4 (5)	95 (59)	**0.45** [0.19; 0.71]	4 (6)	90 (46)	0.07 [– 0.12; 0.27]*	2 (3)	54 (34)
Female	**1.05** [0.13; 1.98]	6 (8)	90 (58)	oEG			**0.55** [0.02; 1.08]	4 (5)	68 (44)	oEG		10 (10)
Training variables												
Training volume (weeks)	*p* = 0.16			*p* = 0.02			*p* = 0.14			*p* = 0.44		
12	**0.87** [0.40; 1.35]	9 (15)	160 (110)	**0.88** [0.19; 1.58]*	2 (3)	30 (20)	**0.40** [0.19; 0.61]	6 (11)	120 (74)	0.43 [– 0.54; 1.39]	3 (4)	54 (44)
21	**0.47** [0.22; 0.72]	2 (3)	62 (40)	0.29 [– 0.53; 1.10]*	3 (3)	72 (46)	**0.62** [0.13; 1.11]	3 (3)	64 (40)	oEG		
Training frequency (sessions/week)	*p* = 0.59			*p* = 0.03			*p* = 0.28			*p* = 0.28		
2	0.56 [0.23; 0.89]	2 (4)	50 (21)	oEG			0.32 [– 0.13; 0.77]	2 (4)	50 (25)	oEG		
3	**0.91** [0.15; 1.67]	8 (10)	113 (90)	oEG			0.46 [– 0.13; 0.77]	4 (5)	62 (42)	0.81 [– 5.40; 7.01]	2 (2)	31 (31)
4	**0.55** [0.25; 0.85]	3 (5)	72 (47)	0.29 [– 0.53; 1.10]*	3 (3)	72 (46)	**0.58** [0.34; 0.81]	3 (5)	72 (47)	oEG		
Session duration (min)	*p* = 0.10			*p* = 0.02			*p* = 0.19			*p* = 0.44		
> 30–60	**0.99** [0.41; 1.57]	9 (12)	115 (98)	**0.88** [0.19; 1.58]*	2 (3)	30 (20)	**0.34** [0.04; 0.65]	5 (7)	55 (54)	0.43 [– 0.54; 1.39]	3 (4)	54 (44)
> 60–90	**0.40** [0.19; 0.61]	3 (5)	91 (52)	0.29 [– 0.53; 1.10]*	3 (3)	72 (46)	**0.61** [0.39; 0.82]	4 (5)	89 (54)	oEG		
Intensity ST (mean)	*p* = 0.03						*p* = 0.90					
Moderate to hard	**0.85** [0.40; 1.31]*	11 (15)	181 (138)	**0.50** [0.04; 0.96]	5 (6)	102 (66)	**0.46** [0.25; 0.68]	8 (10)	130 (94)	0.33 [– 0.31; 0.97]	4 (5)	85 (65)
Intensity ET (mean)	*p* = 0.49			*p* = 0.17			*p* = 0.89			*p* = 0.04		
Low	0.97 [– 0.15; 2.09]	6 (7)	82 (62)	oEG			0.46 [– 0.02; 0.95]	4 (5)	62 (42)	oEG		10 (10)
Moderate to high	**0.64** [0.42;0.87]	7 (12)	153 (96)	0.41 [– 0.02; 0.84]	4 (5)	95 (59)	**0.49** [0.30; 0.68]	5 (9)	122 (72)	0.17 [– 0.14; 0.48]	3 (4)	75 (55)
Training configuration	*p* = 0.51			*p* = 0.10			*p* = 0.12			*p* = 0.66		
Strength prior to endurance	**1.00** [0.02; 1.97]	6 (6)	68 (67)	oEG			0.10 [– 2.70; 2.89]	2 (2)	22 (23)	0.54 [– 1.21; 2.29]	3 (3)	42 (44)
Endurance prior to strength	0.65 [– 0.11; 1.42]	5 (8)	95 (60)	0.97 [– 2.35; 4.28]	2 (2)	18 (20)	**0.49** [0.21; 0.77]	4 (7)	90 (57)	oEG		
Separate days	**0.55** [0.25; 0.85]	3 (5)	72 (47)	0.29 [– 0.53; 1.10]	3 (3)	72 (46)	**0.58** [0.34; 0.81]	3 (5)	72 (47)	oEG		

*CI* confidence interval, *ET* endurance training, *N* total number of participants in the included experimental groups, *N (C)* number of participants in experimental groups (number of participants in control groups), *oEG* only one or no study or experimental group, *S (I)* number of included studies (number of experimental groups), *SMD* weighted standardized mean difference*, ST* strength training

*Significant difference between subgroups

Bold values represent a significant effect (*p* < 0.05)

For muscle power, no effects were observed in adults aged ≤ 65 years (SMD = 0.36 [95% CI − 0.19 to 0.91]; *p* > 0.05; *I*^2^ = 0.0%) and > 65 years (SMD = 0.97 [95% CI − 2.29 to 4.22]; *p* > 0.05; *I*^2^ = 0.0%). In terms of CRE, CT induced moderate effects in adults aged ≤ 65 years (SMD = 0.52 [95% CI 0.35–0.69]; *p* < 0.05; *I*^2^ = 0.0%), with no effects in adults aged > 65 years (SMD = 0.39 [95% CI − 0.27 to 1.05]; *p* > 0.05; *I*^2^ = 0.0%). Additionally, results showed moderate effects in females (SMD = 0.55 [95% CI 0.02–1.08]; *p* < 0.05; *I*^2^ = 0.0%) and small effects in males (SMD = 0.45 [95% CI 0.19–0.71]; *p* < 0.05; *I*^2^ = 0.0%). However, no statistically significant differences between subgroups were noted (*p* > 0.05).

With respect to balance, trivial effects were observed in adults aged ≤ 65 years (SMD = 0.11 [95% CI 0.10–0.11]; *p* < 0.05; *I*^2^ = 0.0%), with no effects in adults aged > 65 years (SMD = 0.54 [95% CI − 1.21 to 2.29]; *p* > 0.05; *I*^2^ = 46.0%). No significant difference between subgroups was noted (*p* > 0.05).

### Results of Single Training Variables Analyses

All results of single training variables analyses are displayed in Table 5. For muscle strength, larger effects of 12 weeks of CT (SMD = 0.87 [95% CI 0.40–1.35]; *p* < 0.05, *I*^2^ = 55.3%) were observed compared with 21 weeks (SMD = 0.47 [95% CI 0.22–0.72]; *p* < 0.05, *I*^2^ = 0.0%). Additionally, results indicated larger effects of three training sessions (SMD = 0.91 [95% CI 0.15–1.67]; *p* < 0.05; *I*^2^ = 71.5%) compared with four sessions per week (SMD = 0.55 [95% CI 0.25–0.85]; *p* < 0.05, *I*^2^ = 0.0%). For session duration, > 30–60 min resulted in larger effects (SMD = 0.99 [95% CI 0.41–1.57]; *p* < 0.05; *I*^2^ = 62.0%) compared with > 60–90 min (SMD = 0.40 [95% CI 0.19–0.61]; *p* < 0.05; *I*^2^ = 0.0%). Similarly, ET of moderate-to-near maximal intensities resulted in moderate effects (SMD = 0.64 [95% CI 0.42–0.87]; *p* < 0.05; *I*^2^ = 0.0%), with no observed effects for low intensities (*p* > 0.05). In terms of training configuration, intra-session ST before ET produced larger effects (SMD = 1.00 [95% CI 0.02–1.97]; *p* < 0.05; *I*^2^ = 63.3%) compared with ST and ET applied on separate days (SMD = 0.55 [95% CI 0.25–0.85]; *I*^2^ = 0.0%). No effects were observed when ET was performed prior to ST within the same session (*p* > 0.05). There was no statistically significant difference between all training variables (*p* > 0.05).

Regarding CRE, larger effects were observed following 21 weeks of CT (SMD = 0.62 [95% CI 0.13–1.11]; *p* < 0.05; *I*^2^ = 0.0%) compared with 12 weeks (SMD = 0.40 [95% CI 0.19–0.61]; *p* < 0.05; *I*^2^ = 0.0%). In addition, four sessions per week induced moderate effects (SMD = 0.58 [95% CI 0.34–0.81]; *p* < 0.05; *I*^2^ = 0.0%), with no effects of three or two sessions per week (*p* > 0.05). For session duration, > 60–90 min resulted in larger effects (SMD = 0.61 [95% CI 0.39–0.82]; *p* < 0.05; *I*^2^ = 0.0%) compared with > 30–60 min (SMD = 0.34 [95% CI 0.04–0.65]; *p* < 0.05; *I*^2^ = 0.0%). ET of moderate-to-near maximal intensities induced small effects (SMD = 0.49 [95% CI 0.30–0.68]; *p* < 0.05; *I*^2^ = 0.0%), while low intensities resulted in no effects (*p* > 0.05). With respect to training configuration, larger effects were observed when ET and ST were conducted on separate days (SMD = 0.58 [95% CI 0.34–0.81]; *p* < 0.05; *I*^2^ = 0.0%) compared with intra-session ET prior to ST (SMD = 0.49 [95% CI 0.21–0.77]; *p* < 0.05; *I*^2^ = 0.0%). No effects were noted following intra-session ST prior to ET (*p* > 0.05). The difference between all training variables was not statistically significant (*p* > 0.05).

For muscle power, results showed that 12 weeks of CT induced large effects (SMD = 0.88 [95% CI 0.19–1.58]; *p* < 0.05; *I*^2^ = 0.0%), while 21 weeks of training resulted in no effects (*p* > 0.05). Of note, the difference between 12 and 21 weeks of training was significant (*p* = 0.016). Regarding session duration, > 30–60 min induced large effects (SMD = 0.88 [95% CI 0.19–1.58]; *p* < 0.05; *I*^2^ = 0.0%), with no effects of > 60–90 min (*p* > 0.05). The difference between subgroups was significant (*p* = 0.016).

### Results of Meta-Regression Analyses

We computed meta-regression for separate training variables (i.e., intervention duration, frequency, CT configuration, session duration, ST intensity, ET intensity) for measures of muscle strength only. Results indicated that none of the training variables predicted the effects of CT on muscle strength (*R*^2^ = 0–3.76%; *p* > 0.05).

## Discussion

The main findings of this study indicated that CT resulted in small-to-moderate effects on measures of physical fitness (i.e., muscle strength, power, and CRE) in middle aged and older adults aged between 50 and 73 years, irrespective of sex. Additionally, the effects of CT on measures of muscle strength and CRE were larger in females compared with males. Results of independent single training factor analysis for different training variables indicated that the largest effects on muscle strength were observed after 12 weeks of training,  > 30–60 min per session, three sessions per week, higher ET intensities and after intra-session ST prior to ET. For CRE, the largest effects were noted after 21 weeks of training, four sessions per week,  > 60–90 min per session, higher ET intensities and after separate ET and ST sessions. Regarding muscle power, the largest effects were observed after 12 weeks of training and with > 30–60 min per session.

### Main Effects

Our results indicate moderate effects of CT on measures of muscle strength and power and small effects on CRE, in agreement with the literature. Several studies indicated that CT produced positive effects on muscle strength [99–101], muscle power [100, 102, 103] and CRE [61, 92, 98] in healthy older adults, regardless of sex. For instance, Wilhelm et al. [88] investigated the effects of 12 weeks (two sessions per week) of CT in 66-year-old men and reported improved muscle strength (i.e., one-repetition-maximum [1RM]; ∆14%), CRE (i.e., $\dot{V}{\text{O}}_{{2{\text{peak}}}}$, ∆7%) and muscle power (i.e., ∆22%). These effects were observed irrespective of the applied exercise sequence (i.e., intra-session ST prior to ET vs. intra-session ET prior to ST). This is supported by Libardi et al. [85], who compared the effects of 12 weeks (two sessions per week) of CT with or without blood flow restriction on CRE, muscle strength and mass in healthy older adults aged 65 years. The authors found that both methods were effective in improving muscle strength (i.e., 1RM; ∆38%) and CRE (i.e., $\dot{V}{\text{O}}_{{2{\text{peak}}}}$; ∆9%). However, we did not find any significant effect of CT on measures of balance. This is not consistent with the general trend in the literature. Earlier studies reported increased balance performance following single-mode strength [104], single-mode aerobic [105] and combined strength and aerobic training [106, 107]. Of note, only four studies were included that measured balance, indicating that this specific outcome should be considered with caution. Nevertheless, the present finding appears to be partly due to an insufficient training stimulus and/or lack of training specificity across the included studies. Results of a systematic review with meta-analysis indicated that balance training protocols are effective to improve balance performance in older adults [108]. Additionally, high-certainty evidence demonstrates that balance exercises mitigate the rate of falls in older adults [10]. As such, to improve balance performance and reduce the rate/risk of falls in older adults, a balance training protocol alongside CT seems to be needed. Taken together, CT is an effective method to enhance measures of physical fitness (i.e., muscle strength, power, balance and CRE) in healthy middle-aged and older adults aged between 50 and 73 years. However, CT appears not to be effective for stimulating improvements in balance performance. This implies that balance exercises in addition to CT seem to be a plausible option to induce gains in balance performance in older adults.

### Subgroup Analyses

Based on our results, moderate and large effects of CT on muscle strength were noted in middle-aged and older adults (i.e., individuals aged ≤ 65 years and > 65 years, respectively). For CRE, only individuals aged ≤ 65 years displayed moderate improvements following CT. The effects of CT on cardiovascular endurance in individuals aged > 65 years were small and statistically non-significant. The latter finding should be considered with caution given the few studies included [84, 86, 88, 89] and the large heterogeneity of the effects across them (SMD = 0.39 [− 0.27 to 1.05]). Takeshima and colleagues [86] reported significant improvements in measures of CRE (e.g., $\dot{V}{\text{O}}_{{2{\text{peak}}}}$) and muscle strength (e.g., knee flexors) in healthy older adults aged 68 years following 12 weeks of CT (three sessions per week). Likewise, Haykowsky et al. [84] compared the effects of 12 weeks (three sessions per week) of CT, ET and ST versus a control group on CRE (i.e., $\dot{V}{\text{O}}_{{2{\text{peak}}}}$) and muscle strength (i.e., 1RM leg press and chest press) in healthy older women aged a mean 68 years. The authors found that CT is as effective as ET and ST alone to improve CRE and muscle strength.

In terms of sex, our findings indicated larger effects of CT on measures of muscle strength and CRE in females compared with males. However, the difference between subgroups was not statistically significant. Similar to our findings, larger increases in muscle strength and cardiovascular endurance in older females compared with older males were observed in previous studies [109, 110]. In a systematic review with meta-analysis of sex differences in adaptations following ST in older adults, Jones et al. [109] revealed larger relative improvements in lower limb muscle strength in females compared with males. However, when gains are expressed in absolute terms, older males showed larger improvements in upper and lower limb strength than older females. This indicates that the interpretation of sex-related adaptation differences is dependent on the way the results are presented (i.e., absolute vs. relative). Of note, despite the absolute expression of older females’ outcomes in this study, we were able to observe larger gains compared with older males. Similarly, a relatively higher increase in cardiovascular endurance (i.e., maximal oxygen uptake [$\dot{V}{\text{O}}_{2\max }$]) was reported in older females (∆22%) compared with older males (∆19%) following ET [110]. Multiple factors could underpin the difference in the magnitude of response to training between males and females, among which is training status. However, we were not able to gain consistent information about participants’ training status due to the lack of relevant details in the included studies. It is worth noting though that none of the included studies directly contrasted the effects of CT between males and females, highlighting a gap in the literature. Because our findings are based on an indirect comparison between studies, they have to be interpreted with caution. Future studies that directly contrast the effects of CT between males and females and across different older adults’ age groups are needed.

### Results of Single Training Variable Analyses

The largest effects of CT on muscle strength and power were observed after 12 weeks. However, for CRE, the largest improvements were noted after 21 weeks of training. This indicates different time-course-specific adaptations between muscle strength/power and CRE, being shorter in the former compared with the latter. Karavirta et al. [79] investigated the effects of 21 weeks of CT on measures of CRE (i.e., $\dot{V}{\text{O}}_{2\max }$) and muscle strength (i.e., knee extension) in healthy untrained men aged a mean 54 years. The authors reported marked improvement in CRE after 21 weeks of training. However, it is worth mentioning that the improvements in V̇O_2max_ followed a gradual pattern throughout the entire program. More specifically, the authors reported that CT lasting > 12 weeks induced larger gains than CT lasting ≤ 12 weeks. Additionally, our results indicated that longer single CT session durations (i.e., > 60–90 min) produced the largest enhancements in CRE. In contrast, the largest effects on muscle strength and power were observed after shorter session durations (i.e., > 30–60 min). Taken together, unlike muscle strength and power, it seems that longer duration of total training as well as of single training sessions is key to stimulating substantial improvements in CRE in middle-aged and older adults.

With respect to CT frequency, the largest effects on muscle strength were observed after three sessions per week. For CRE, results indicated that four sessions per week induced the largest adaptations. Of note, the frequency of training has an effect on the weekly distribution of intervention duration [111]. Therefore, this particular finding is consistent with the outcomes related to total training duration and single-session duration. More specifically, our analyses indicated that more frequent exposure to CT with its potential effects on the weekly distribution of intervention duration benefits CRE more than muscle strength. Ferrari et al. [112] examined the effects of 10 weeks of different weekly CT frequencies (i.e., two vs. three sessions) on CRE in previously trained, healthy older men aged 65 years. The authors found similar effects on $\dot{V}{\text{O}}_{{2{\text{peak}}}}$ following both training frequencies. However, for maximum aerobic workload, significant improvements were reported only after three CT sessions per week. Taken together, it seems that CRE and muscle strength are differentially influenced by CT frequency, suggesting that higher frequency benefits the former more than the latter.

Regarding training intensity, our findings indicate that moderate-to-near maximal ET intensities benefit both muscle strength and CRE more than low intensities do. This implies that to optimize CT-related strength and cardiorespiratory adaptations, higher ET intensities should be favored. It has previously been shown that higher ET intensities (≥ lactate threshold intensity) resulted in larger CRE gains in young adults aged between 18 and 35 years compared with lower intensities [113]. In older adults, larger CRE adaptations following higher compared with lower ET intensities were reported [81]. Fyfe et al. [114] investigated the effects of two different ET intensities (i.e., moderate vs. high intensity) prior to ST on the mechanistic target of rapamycin complex 1 (mTORC1) signaling in healthy young adults aged a mean 27 years. The authors found that neither of the applied intensities attenuated the mTORC1 signaling pathway. They also showed that high-intensity ET may provide a greater anabolic stimulus compared to moderate ET intensities, suggesting it is a facilitator for strength adaptations. On the other hand, it has been argued that higher intensities of ET can lead to metabolic perturbation in type II muscle fibers (e.g., glycogen depletion), reducing anabolic responses to ST [115, 116]. Clearly, the outcomes of the literature as to ET intensities and their effects on the underpinning mechanisms of muscle strength adaptation are heterogeneous, precluding any consistent conclusion. This is why future studies are needed to further clarify this aspect.

Exercise configuration during CT is another aspect that requires attention as it affects the magnitude of muscle strength [117, 118] and CRE [119] adaptations. Currently, there is no consensus concerning the most effective exercise configuration [119]. However, CT configuration recommendations are mainly based on the program priorities/desired adaptation (i.e., muscle strength or CRE). Our findings indicated a larger increase in muscle strength when ST preceded ET. As such, to optimize muscle strength adaptations, intra-session ST prior to ET should be favored. This is in agreement with the literature [117–119]. Cadore et al. [120, 121] reported that performing ST prior to ET is the optimal sequence to induce muscle strength adaptations in older adults aged 65 years. Eddens et al. [117] conducted a systematic review with meta-analysis and provided evidence for the favorable effects of intra-session ST prior to ET on lower limb muscle strength adaptations in healthy adults aged between 18 and 65 years. This same exercise sequence was supported by the meta-analytical study of Murlasits et al. [118]. Of note, our findings indicated no significant effects on muscle strength when ET was applied prior to ST. This seems to be due to ET-inducing residual fatigue, which may hinder training-induced muscle strength gains [120]. For CRE, while intra-session ET prior to ST produced small effects, ET and ST applied on separate days resulted in moderate effects, suggesting favorable effects of the latter. Of note, intra-session ST prior to ET produced no effects on CRE. Generally, it has been reported that the sequence ET prior to ST is the better choice for developing maximal aerobic power [119]. Additionally, separation of sessions has been suggested by earlier studies as a useful strategy to optimize CT adaptations [118]. However, an earlier meta-analysis indicated that intra-session CT sequence has no impact on CRE [118]. Of note, the meta-analysis of Murlasits et al. [118] included trials that considered both sexes and various age groups (i.e., from 14 to 66 years), with only four studies conducted with older adults. The same applies to the meta-analysis of Eddens et al. [117], where two studies only out of the ten included addressed older adults. Our meta-analysis, however, attempted to overcome the previous limitations by focusing on middle-aged and older adults only. Overall, practitioners are advised to manipulate exercise configuration during CT according to the desired adaptation. Specifically, ST prior to ET appears to be an adequate sequence to optimize muscle strength in middle-aged and older adults. For larger CRE gains, ET and ST on separate days should be prioritized over intra-session ET prior to ST.

### Limitations and Future Research Perspectives

Our results were based on studies that investigated the effects of CT on measures of physical fitness (i.e., muscle strength and CRE), with no emphasis on the mechanistic aspects (e.g., key pathways of muscle protein synthesis). Of note, there is a dearth of data in the current literature on the underlying physiological mechanisms. Further, while the main outcomes of the present analysis are relevant from a practical standpoint, it would have also been interesting to meta-analyze the effects of CT versus single-mode ST or ET in middle-aged and older adults. This should be addressed in the future. Additionally, some of the addressed outcome measures displayed moderate-to-substantial heterogeneity. Indeed, we accounted for the amount of heterogeneity by applying a random-effects model and constantly reporting *I*^2^ values of the respective outcome. Also, moderator analyses represent an additional useful tool to explore heterogeneity across different subgroups or training variables to estimate effect specific to the respective group or variable [68, 122–124]. However, we should acknowledge that the small number of studies included in subgroup analyses often provides insufficient statistical power, inflating the risk of type II error rate [68, 125]. Moreover, moderator analyses were computed independently, ignoring any potential interaction between variables. Therefore, the results of moderator analyses must be considered with caution, though we do consider the current analyses as an appropriate starting point to establish effective dose–response relationships of the effects of CT on measures of physical fitness in middle-aged and older adults. Finally, the PEDro score of most of the included studies is below the cut-off value of 6, reflecting low methodological quality and high risk of bias (Table 4). It is, however, worth noting that blinding of participants and investigators is not feasible in exercise interventions. Also, blinding of assessors is rarely considered. In this sense, none of the included studies considered the blinding of participants or investigators, while only four of them considered blinding of assessors, increasing the risk of bias in the reported outcomes. Therefore, future studies with higher methodological quality are warranted.

## Conclusion

CT is an effective method to improve measures of physical fitness (i.e., muscle strength, power, and CRE) in healthy adults aged between 50 and 73 years, irrespective of sex. Therefore, CT is recommended for middle-aged and older adults to maintain/improve functional capacities and promote health. Additionally, results of independent single training factor analysis indicated that 12 weeks of training, > 30–60 min per session, three sessions per week, higher ET intensities and intra-session ST prior to ET produced the largest effects on muscle strength. For muscle power, the largest effects were observed after 12 weeks of training and > 30–60 min per session. Regarding CRE, the largest effects were observed after 21 weeks of training, four sessions per week, > 60–90 min per session, higher ET intensities and after separate ET and ST sessions. Practitioners can use CT to improve physical fitness (i.e., muscle strength, power, and CRE) in middle-aged and older adults. Moreover, results of independent single training factor analysis can serve to guide CT prescription in middle-aged and older adults.

## Declarations

### Availability of data and material

The data and R code and analysis documentation are freely available on repositories of the Open Science Framework (https://osf.io/3xd7p/).
